# Site-specific contacts enable distinct modes of TRPV1 regulation by the potassium channel Kvβ1 subunit

**DOI:** 10.1074/jbc.RA120.015605

**Published:** 2020-10-15

**Authors:** Yuanyuan Wang, Xiaoyi Mo, Conghui Ping, Qian Huang, Hao Zhang, Chang Xie, Bo Zhong, Dongdong Li, Jing Yao

**Affiliations:** 1 State Key Laboratory of Virology, Hubei Key Laboratory of Cell Homeostasis, College of Life Sciences, Frontier Science Center for Immunology and Metabolism, Wuhan University, Wuhan, Hubei, China; 2 Sorbonne Université, Institute of Biology Paris Seine, Neuroscience Paris Seine, Sorbonne Université, Paris, France

**Keywords:** Kvβ1 subunit, nociception, protein–protein interaction, TRPV1, pain, thermal sensation, transient receptor potential channels (TRP channels), dorsal root ganglia, electrophysiology, calcium, patch clamp, physiology, Kvβ1 subunit

## Abstract

Transient receptor potential vanilloid 1 (TRPV1) channel is a multimodal receptor that is responsible for nociceptive, thermal, and mechanical sensations. However, which biomolecular partners specifically interact with TRPV1 remains to be elucidated. Here, we used cDNA library screening of genes from mouse dorsal root ganglia combined with patch-clamp electrophysiology to identify the voltage-gated potassium channel auxiliary subunit Kvβ1 physically interacting with TRPV1 channel and regulating its function. The interaction was validated *in situ* using endogenous dorsal root ganglia neurons, as well as a recombinant expression model in HEK 293T cells. The presence of Kvβ1 enhanced the expression stability of TRPV1 channels on the plasma membrane and the nociceptive current density. Surprisingly, Kvβ1 interaction also shifted the temperature threshold for TRPV1 thermal activation. Using site-specific mapping, we further revealed that Kvβ1 interacted with the membrane-distal domain and membrane-proximal domain of TRPV1 to regulate its membrane expression and temperature-activation threshold, respectively. Our data therefore suggest that Kvβ1 is a key element in the TRPV1 signaling complex and exerts dual regulatory effects in a site-specific manner.

TRPV1 is a Ca^2+^-permeable cation channel and plays important roles in pain sensation and transduction. It is abundantly expressed in sensory neurons in the dorsal root ganglia (DRG) and trigeminal ganglia (1). As a multimodal sensor, TRPV1 responds to a variety of physical or chemical stimuli such as heat (>42 °C), voltage, low pH, capsaicin, and analogs like nonivamide and resiniferatoxin (1–3). During injury and inflammation, inflammatory mediators (*e.g.* bradykinin, nerve growth factor) enhance the responses of TRPV1 to noxious stimuli, thereby heightening pain experience, a process called hyperalgesia. TRPV1 knockout mice show impaired thermal nociception and vanilloid-induced pain (4, 5).

TRPV1 appears usually as a homotetramer channel and also forms heterotetramers with other TRP family members (6, 7). At the channel level, TRPV1 function is delicately controlled by multiple mechanisms including protein kinase–mediated intrachannel phosphorylation or SUMOylation (8–10) and subcellular trafficking (11–14). In addition, because ion channels are organized in multiprotein assemblies, termed signaling complexes, channel activity is also regulated by local protein–protein interactions (15). TRPV1 function has been suggested to be influenced by a variety of proteins. For example, GABA_A_ receptor–associated protein is implicated in TRPV1 clustering on the plasma membrane, a process relying on the intermediate interaction with tubulin (16). Cyclin-dependent kinase 5 was reported to alter TRPV1 membrane transport via the involvement of kinesin-3 family member 13B protein (17). However, the molecular components directly interacting with TRPV1 channels remain to be determined.

Here, we screened a cDNA library of mouse DRG by yeast two-hybrid assay and identified the voltage-gated K^+^ channel β1 subunit (Kvβ1) directly interacting with TRPV1 protein. By patch-clamp recording, Kvβ1 was observed to regulate the temperature threshold for TRPV1 thermal activation and the whole-cell current density. Mechanistically, Kvβ1 played a role in sustaining the availability of TRPV1 on the plasma membrane. Site-specific mapping identified that Kvβ1 interacted with the membrane-proximal domain (MPD) and membrane-distal domain (MDD) of TRPV1 to orchestrate its temperature-activation threshold and surface expression, respectively. These data suggest that Kvβ1 subunit exerts dual regulatory effects on TRPV1 function and represents a functional component in the signaling complex. Targeting Kvβ1–TRPV1 interaction would help to regulate nociceptive transduction.

## Results

### Kvβ1 physically interacts with TRPV1 channel

**Figure 1.**
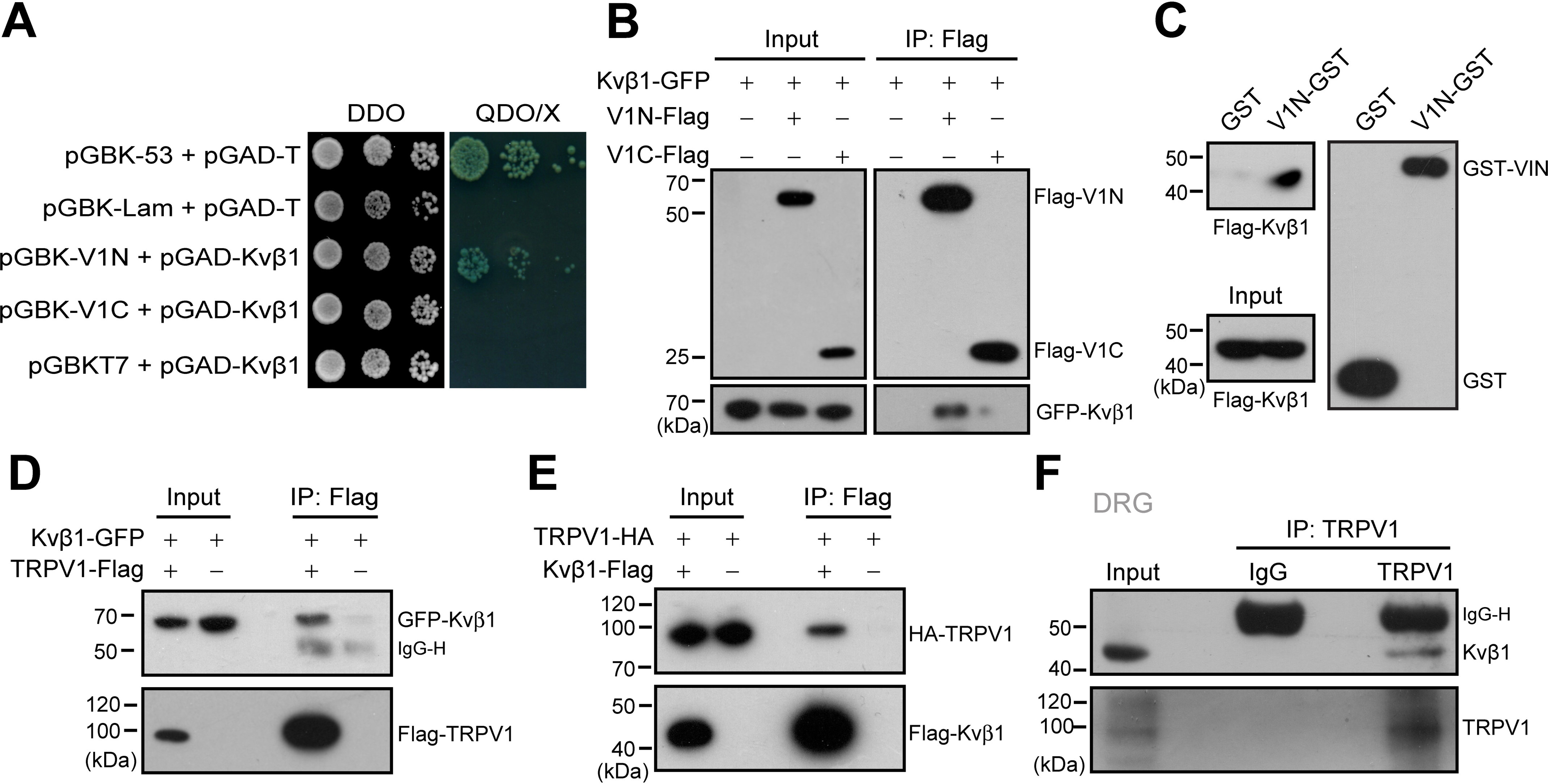
**Kvβ1 physically interacts with TRPV1 channel.** *A*, yeast two-hybrid results showing the interaction between Kvβ1 and the TRPV1 N terminus (*V1N*) rather than the C terminus (*V1C*). The 53-T and Lam-T were served as positive and negative controls, respectively. *DDO*, double dropout medium (SD/−Leu/−Trp); *QDO*, quadruple dropout medium (SD/−Leu/−Trp/−His/−Ade); *X*, X-α-gal. *B*, IP and IB analysis of HEK 293T cells transfected with plasmids encoding FLAG-tagged TRPV1-Nt, TRPV1-Ct, and GFP-tagged Kvβ1. Cell lysates were IP with FLAG antibody and analyzed by IB using anti-GFP and anti-FLAG, respectively. Whole-cell lysates were also used for IB with anti-GFP and anti-FLAG as input. *C*, GST pulldown analysis of Kvβ1 with GST fusion N-terminal of TRPV1 (V1N-GST). GST-fusion proteins encompassing TRPV1 N terminus were constructed and expressed in *E. coli* strain BL21. The TRPV1 N terminus fusion protein bound to GSH-Sepharose beads was then incubated with HEK 293T cell lysate. GST only was used as a negative control. *D* and *E*, HEK 293T cells were transfected with the indicated plasmids. Immunoprecipitation (with anti-FLAG) and immunoblot analysis (with anti-GFP and anti-FLAG in *D* or anti-HA and anti-FLAG in *E*) of the interaction between TRPV1 and Kvβ1. *F*, immunoprecipitation (with anti-TRPV1 or IgG as a control) and immunoblot analysis (with anti-TRPV1 or anti-Kvβ1) of DRG neurons. The data are representative of three independent experiments. Full images of the Western blots are shown in Fig. S4.

In an attempt to identify the protein that directly interact with TRPV1 channel in DRG neurons, a yeast two-hybrid assay was performed to screen a mouse DRG cDNA library while using the cytosolic N-terminal domain of mouse TRPV1 as the bait. Sequence analysis of the positive clones revealed that 18 proteins interacted with the N terminus of TRPV1 (V1N). Among them, Kvβ1, the auxiliary subunit of voltage-gated potassium channels showed strong interaction with TRPV1. To directly verify this interaction, we co-transformed Kvβ1-pGADT7 with V1-Nt-pGBKT7 or V1-Ct-pGBKT7 into the yeast reporter strain AH109. Indeed, Kvβ1 interacted with the V1N, but not the C terminus (V1C) of TRPV1 (Fig. 1*A*). Then the co-immunoprecipitation (co-IP) experiment was conducted to examine Kvβ1–TRPV1 interaction in HEK 293T cells. As shown in Fig. 1*B*, Kvβ1–GFP was co-immunoprecipitated by V1-Nt-FLAG, but not V1-Ct-FLAG or the FLAG vector alone, confirming that Kvβ1 interacts with the N terminus of TRPV1 rather than the C terminus.

Because co-IP experiment cannot exclude the possibility of indirect protein–protein interaction with accessory proteins, we carried out the gluatathione *S*-transferase (GST) pulldown experiment to examine the direct interaction between Kvβ1 and the N terminus of TRPV1. We generated the GST-fusion protein containing the N terminus of TRPV1 (named V1N-GST). Then V1N-GST and GST were immobilized on GSH-Sepharose beads and incubated with the HEK 293T extracts that expressed FLAG-Kvβ1. As illustrated in Fig. 1*C*, V1N-GST but not GST specifically retained the recombinant Kvβ1, thus corroborating the yeast two-hybrid screen and co-IP results. Next, we evaluated the capacity of Kvβ1 to bind the full length of TRPV1 channel in HEK 293T cells using a co-IP strategy. As shown in Fig. 1 (*D* and *E*), Kvβ1-GFP could be co-immunoprecipitated by TRPV1–FLAG with FLAG-agarose beads. Reciprocally, TRPV1-HA could be precipitated by Kvβ1-FLAG. These results showed that Kvβ1 and TRPV1 interact with each other *in situ* in HEK 293T cells. Further, the interaction between Kvβ1 and TRPV1 was validated with co-IP assay in DRG neurons. We homogenized mouse DRGs and observed the co-precipitation of the endogenous TRPV1 and Kvβ1 proteins by using the specific antibodies (Fig. 1*F*). Together, Kvβ1 and TRPV1 are physically associated in native DRG neurons.

### Kvβ1 up-regulates TRPV1 current responses

**Figure 2.**
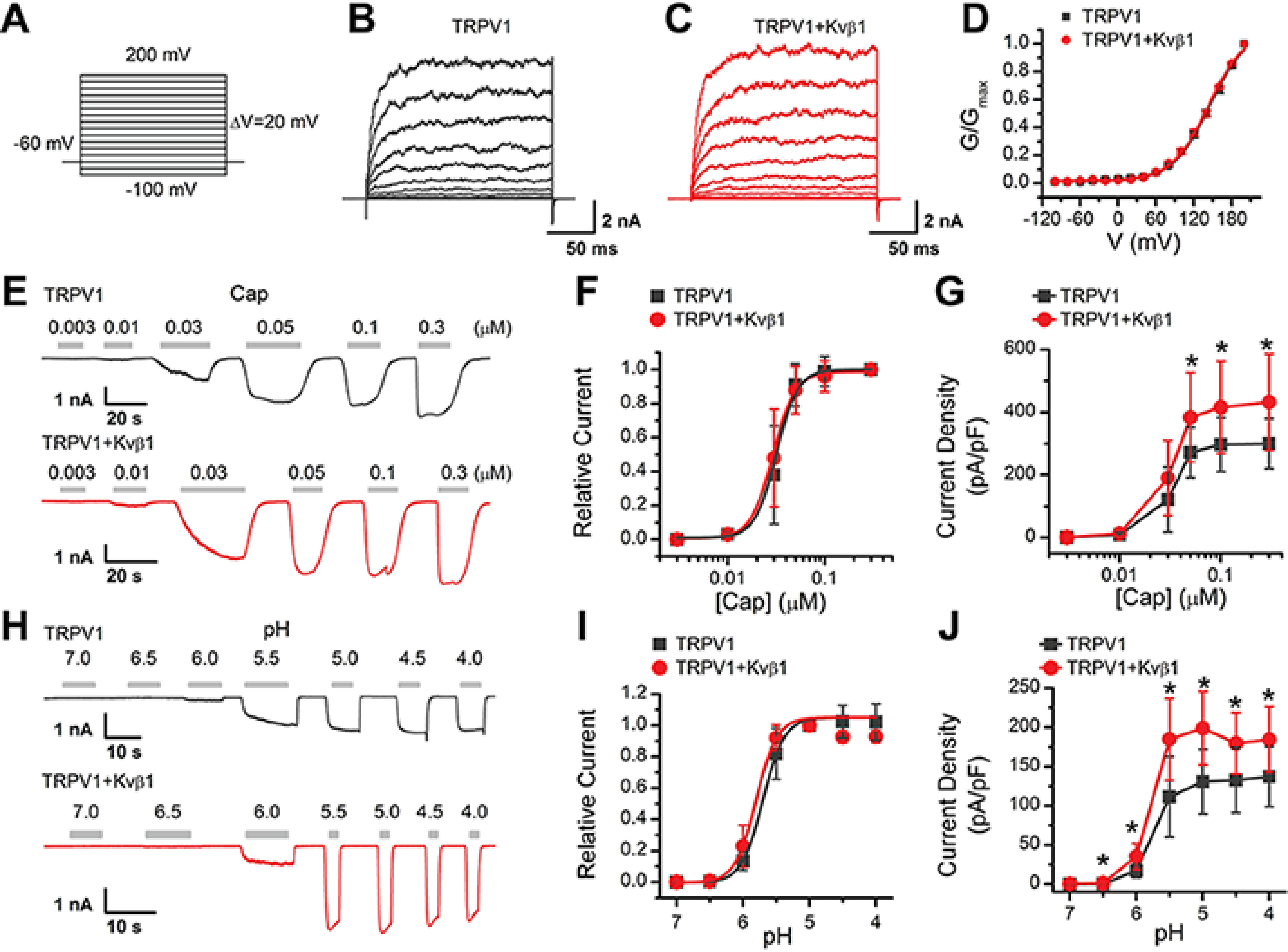
**Kvβ1 enhances TRPV1 activity.** *A*, voltage protocol with 200-ms test pulses ranging from −100 to +200 mV in 20-mV increments from holding potential of −60 mV. *B* and *C*, representative whole-cell recordings in HEK 293T cells expressing TRPV1 alone (*B*) and TRPV1 + Kvβ1 (*C*). TRPV1 currents were elicited by the voltage protocol as *A*. *D*, normalized conductance–voltage (*G*–*V*) relations. Boltzmann fits correspond to *V*_1/2_ = 155.8 ± 1.9 mV, and κ = 34.6 ± 0.9 for TRPV1 (*n* = 6), and *V*_1/2_ = 153.5 ± 1.9 mV, and κ = 34.1 ± 1.0 for TRPV1 + Kvβ1 (*n* = 9), respectively. *E*, representative whole-cell recordings from HEK 293T cells that expressed TRPV1 (*top panel*) and TRPV1 + Kvβ1 (*bottom panel*). The cells were exposed to varied concentrations of capsaicin (*Cap*) as indicated. The holding potential was −60 mV. *F*, concentration-response curves for capsaicin-evoked currents. *Solid lines* indicate fits with the Hill equation, which yielded EC_50_ = 0.03 ± 0.01 µm and *n*_H_ = 3.9 ± 0.2 for TRPV1 (*n* = 12) and EC_50_ = 0.03 ± 0.01 μm and n_H_ = 3.8 ± 0.4 for TRPV1 + Kvβ1 (*n* = 11). *G*, summary plot of current density for TRPV1 (*n* = 12) and TRPV1 + Kvβ1 (*n* = 11). The peak current densities evoked by capsaicin are normalized by membrane capacitance. *, *P* < 0.05. *H*, parallel recordings of H^+^-evoked responses in transiently transfected HEK 293T cells held at −60 mV. *I*, dose-response curves of low pH. The *solid lines* are fits to Hill's equations with pH_50_ = 5.70 ± 0.02 and n_H_ = 2.7 ± 0.3 for TRPV1 (*n* = 7) and pH_50_ = 5.80 ± 0.07 and n_H_ = 2.8 ± 0.8 for TRPV1 + Kvβ1 (*n* = 7). *J*, comparison of current density evoked by pH 5.0 between TRPV1 (*n* = 7) and TRPV1 + Kvβ1 (*n* = 7). *, *P* < 0.05. Holding potential was −60 mV. *Error bars* represent S.D.

Because HEK 293T cells are commonly used as an expression system for exogenous ion channel and they lack endogenous Kvβ1 expression (Fig. S1), we next investigated the effects of Kvβ1 on TRPV1 activity by co-expressing both components in this cell line. Kvβ1, as an auxiliary subunit of the voltage-gated potassium channel, regulates the rapid inactivation of Kv1 family channels (18). Because TRPV1 can be activated by membrane depolarization, we examined the effect of Kvβ1 on the voltage-dependent TRPV1 currents using a voltage step protocol (Fig. 2*A*). Whole-cell currents were elicited with 200-ms depolarizing pulses ranging from −100 mV to 200 mV with 20-mV increments in HEK 293T cells that expressed either TRPV1 alone or TRPV1 and Kvβ1 (Fig. 2, *B* and *C*). The current at the end of the 200-ms pulse was converted to conductance and normalized to that obtained at 200-mV stimulation. Subsequent fitting with the Boltzmann function yielded similar conductance–voltage (*G*–*V*) relationships for TRPV1 response regardless of whether Kvβ1 was present or not (Fig. 2*D*). We observed no currents in HEK 293T cells solely expressing Kvβ1 (Fig. S2). Thus, co-expression of Kvβ1 did not change the voltage dependence of TRPV1 under basal conditions.

Then we examined the effect of Kvβ1 on TRPV1 channel gating as evoked by capsaicin or acidic extracellular solutions. We compared TRPV1 function by activating the channel with increasing concentrations of capsaicin at the holding potential (*V*_h_) of −60 mV. The amplitude of the evoked currents was normalized to that evoked by 0.3 μm capsaicin. Fitting the dose-response curves with the Hill equation yielded similar EC_50_ values and Hill coefficients (*n*_H_) for TRPV1 co-expressed with Kvβ1 or not (Fig. 2, *E* and *F*), EC_50_ = 0.03 ± 0.01 μm, *n*_H_ = 3.9 ± 0.2 for TRPV1 and EC_50_ = 0.03 ± 0.01 μm, *n*_H_ = 3.8 ± 0.4 for TRPV1 + Kvβ1). The maximum current density, however, showed a significant difference between the absence (300.9 ± 79.4 pA/pF, *n* = 12) and the presence of Kvβ1 (432.5 ± 153.5 pA/pF, *n* = 11; Fig. 2*G*). We also investigated TRPV1 responses to protons by applying variable acidic solutions covering a broad pH range from 7.0 to 4.0 and determined pH_50_ and *n*_H_. Akin to capsaicin induced gating, co-expression of Kvβ1 with TRPV1 did not alter the concentration dependence to proton but increased the current density, *e.g.* 130.8 ± 41.2 pA/pF (*n* = 7) and 199.0 ± 46.8 pA/pF (*n* = 7) evoked by pH 5.0 for TRPV1 and TRPV1 + Kvβ1, respectively (Fig. 2, *H–J*). Taken together, these results show that Kvβ1 modulates whole-cell current density of TRPV1.

### Kvβ1 sustains the surface expression of TRPV1

**Figure 3.**
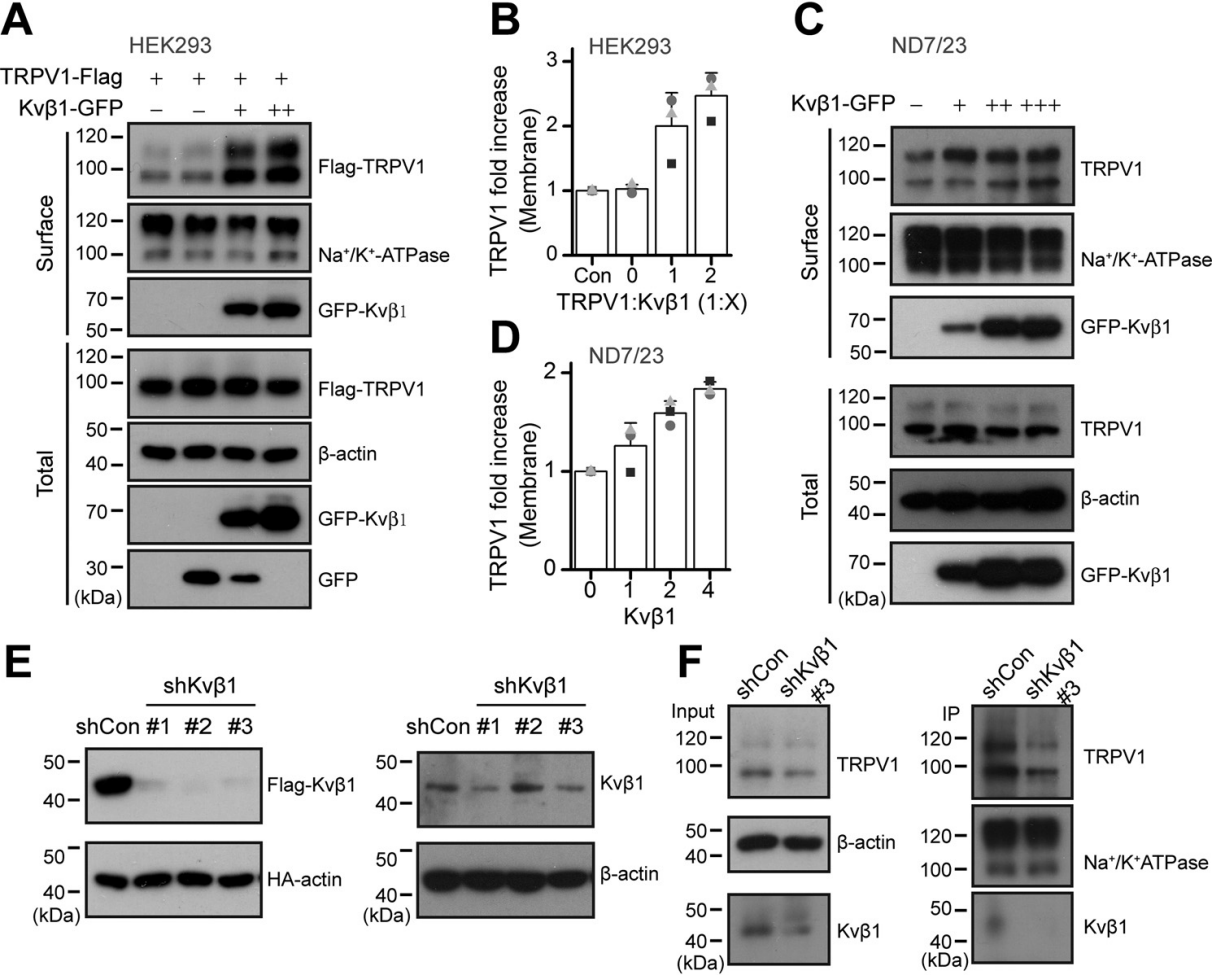
**Kvβ1 sustains the surface expression of TRPV1.** *A*, increased surface expression of TRPV1 by Kvβ1. HEK 293T cells were transiently co-transfected with different molar ratios of TRPV1-FLAG and Kvβ1-GFP as indicated. Note that the amount of TRPV1-FLAG was fixed. Surface levels of TRPV1 were measured by IB after plasma membrane proteins were biotinylated and purified with NeutrAvidin-agarose beads. β-Actin and Na^+^/K^+^ ATPase were used as controls for cytoplasmic and membrane proteins, respectively. *B*, quantitative analysis of the fold increase of TRPV1 on the plasma membrane following co-expression of Kvβ1 in HEK 293T cells (*n* = 3; means ± S.D.). *C* and *D*, ND7/23 cells were transiently transfected with increasing amounts of Kvβ1-GFP cDNA. Similarly, surface levels of TRPV1 were measured by IB after plasma membrane proteins were biotinylated and purified with NeutrAvidin-agarose beads (*n* = 3; means ± S.D.). *E*, immunoblot analysis (with anti-FLAG or anti-HA) of HEK 293T cells transfected for 36 h with plasmids encoding FLAG-tagged Kvβ1 and HA-β-actin and either Kvβ1-targeting shRNA (shKvβ1#1, shKvβ1#2, and shKvβ1#3) or shCon to test knockdown efficiency of shRNA (*left panel*). Immunoblot analysis (with anti-Kvβ1 or anti-β-actin) of ND7/23 cells stably transfected with shCon, shKvβ1#1, shKvβ1#2, or shKvβ1#3 (*right panel*). *F*, the cell-surface biotinylation assay was used to measure the surface levels of TRPV1 in ND7/23 cells that were stably transfected with shCon or shKvβ1#3. Full images of the Western blots are shown in Fig. S5.

Because the presence of Kvβ1 increased TRPV1 current density without altering its agonist sensitivity, we reasoned that Kvβ1 might have increased TRPV1 surface expression level. To test this hypothesis, we performed a surface biotinylation experiment. As shown in Fig. 3 (*A* and *B*), in HEK 293T cells, Kvβ1 proportionally increased the surface expression but not the total amount of TRPV1 channel.

We further used the ND7/23 neuroblastoma cell line that is a hybridization of mouse neuroblastoma and rat DRG neuron (19). This immortal cell line inherits the endogenous TRPV1 expression of DRG neurons. Then we explored the effect of overexpressed Kvβ1 subunit on TRPV1 protein expression in the ND7/23 cell line. As shown in Fig. 3*C*, along with the amount of transfected Kvβ1 increased, the expression level of TRPV1 on the plasma membrane was proportionally up-regulated. Transfection of 4 μg of Kvβ1 caused a nearly 2-fold increase TRPV1 plasma membrane expression (Fig. 3*D*). In this condition, however, the total amount of TRPV1 was unaffected. Next, we evaluated the regulatory effect of Kvβ1 on TRPV1 expression using shRNA-mediated knockdown. To test the knockdown efficiency of three groups of shRNAs designed against Kvβ1, we co-transfected them or empty vector shCon with FLAG-Kvβ1 and HA-actin into HEK 293T cells, respectively. HA-actin was used to calibrate the transfection efficiency between groups. The expression of the two proteins were evaluated by immunoblotting. All the three shRNAs efficiently reduced Kvβ1 protein expression levels (Fig. 3*E*). We also validated the inhibitory efficiency of the shRNAs in ND7/23 cells that express Kvβ1 subunit, although at a relatively low level (Fig. S1). As shown in the *right panels* of Fig. 3*E*, shKvβ1#1 and shKvβ1#3 both inhibited the endogenous expression of Kvβ1. We then performed surface biotinylation experiment in ND7/23 cells to detect the impact of shKvβ1#3 on the total and surface expression of TRPV1. We observed that inhibition of Kvβ1 expression concomitantly reduced the expression of TRPV1 on the cell surface (Fig. 3*F*, *right panels*). These results together suggest that Kvβ1 sustains the functional expression of TRPV1 on the plasma membrane.

### Molecular domains underlying the association of Kvβ1 and TRPV1

**Figure 4.**
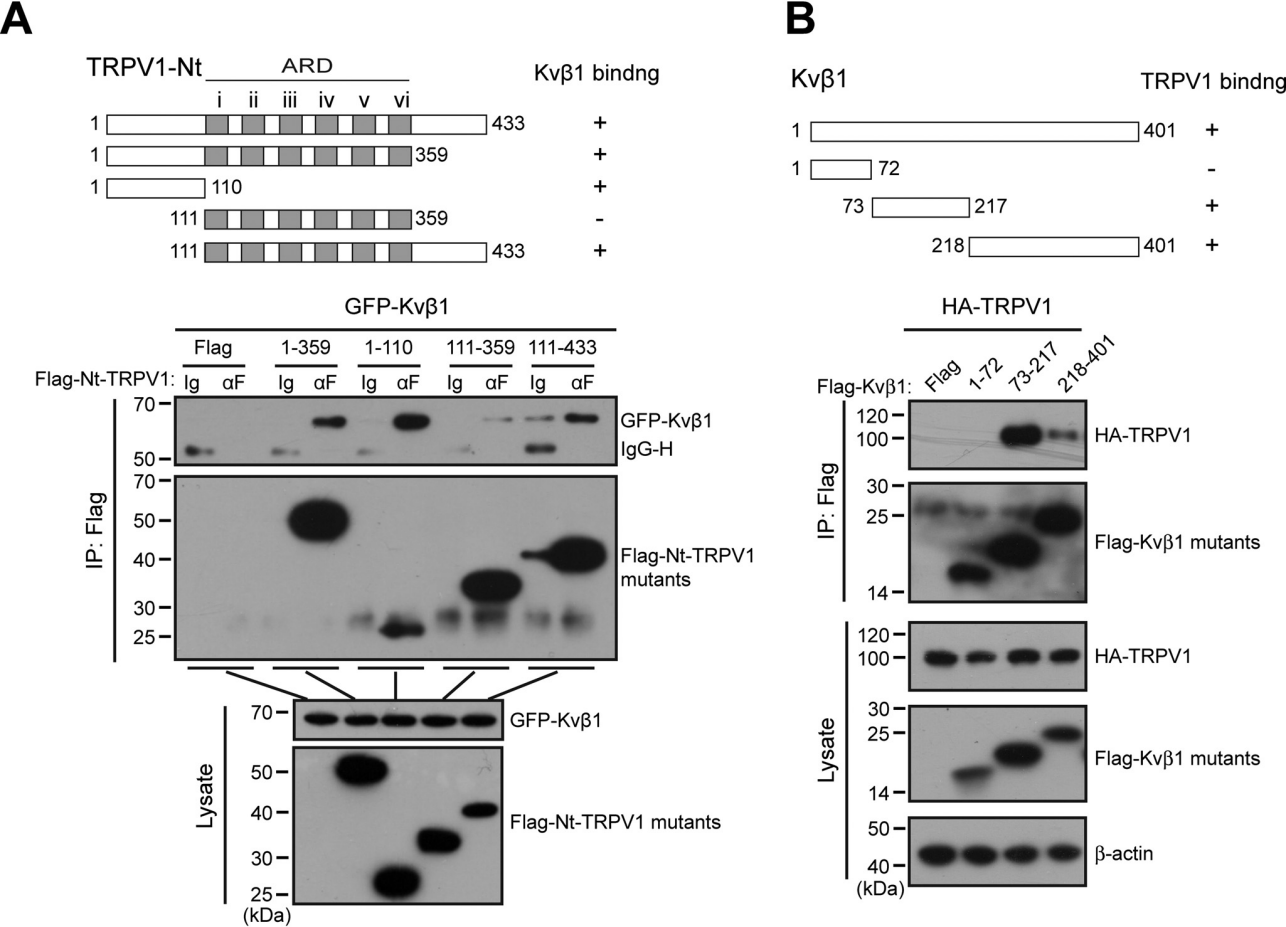
**Two distinct domains mediate the interaction between the N terminus of TRPV1 and Kvβ1.** *A*, schematic representation of the truncation mutants of N terminus of TRPV1 including 1–359, 1–110, 111–359, and 111−433 segments, respectively. HEK 293T cells were transiently co-transfected with different truncation mutants tagged with FLAG and Kvβ1-GFP as indicated. Cell lysates were immunoprecipitated with anti-FLAG and analyzed by IB using anti-GFP and anti-FLAG, respectively. Whole-cell lysates were also used for IB with anti-GFP and anti-FLAG as input. *B*, schematic diagram of composition of mutant Kvβ1 including the 1–72, 73–217, and 218–401 segments. HEK 293T cells were transfected with TRPV1-HA and different mutant Kvβ1 fused with FLAG as indicated. The cell lysates were subjected to IP by anti-FLAG, followed by IB for anti-HA and anti-FLAG, respectively. All blot images are representatives of three independent experiments. Full images of the Western blots are shown in Fig. S6.

To define the critical domains within the N terminus of TRPV1 that mediate Kvβ1-TRPV1 association, a series of Nt-TRPV1 truncations were constructed, including FLAG-1–359, FLAG-1–110, FLAG-111–359, and FLAG-111–433 (Fig. 4*A*). They were individually co-expressed with Kvβ1-GFP in HEK 293T cells for co-IP evaluation. Kvβ1-GFP was strongly precipitated by FLAG-1–110, as was FLAG-1–359 and FLAG-111–433 but not FLAG-111–359 (Fig. 4*A*). These data indicate that both the MDD (aa 1–110) and MPD (aa 359–433) of TRPV1 channel are required for associating with Kvβ1.

We further mapped the molecular domains of Kvβ1 that mediated its interaction with TRPV1. Three truncations were constructed, including FLAG-1–72, FLAG-73–217, and FLAG-218–401. In a yeast two-hybrid experiment, the aa 218–401 segment had been noted to mediate the interaction between Kvβ1 and TRPV1 N terminus and therefore was used as a positive control. As shown in Fig. 4*B*, TRPV1-HA could be precipitated by both the middle (aa 73–217) and ending part (aa 218–401) of Kvβ1, suggesting that the segment between aa 73 and 401 of Kvβ1 interacts with TRPV1.

### Kvβ1 interacts with MDD to regulate TRPV1 surface expression

**Figure 5.**
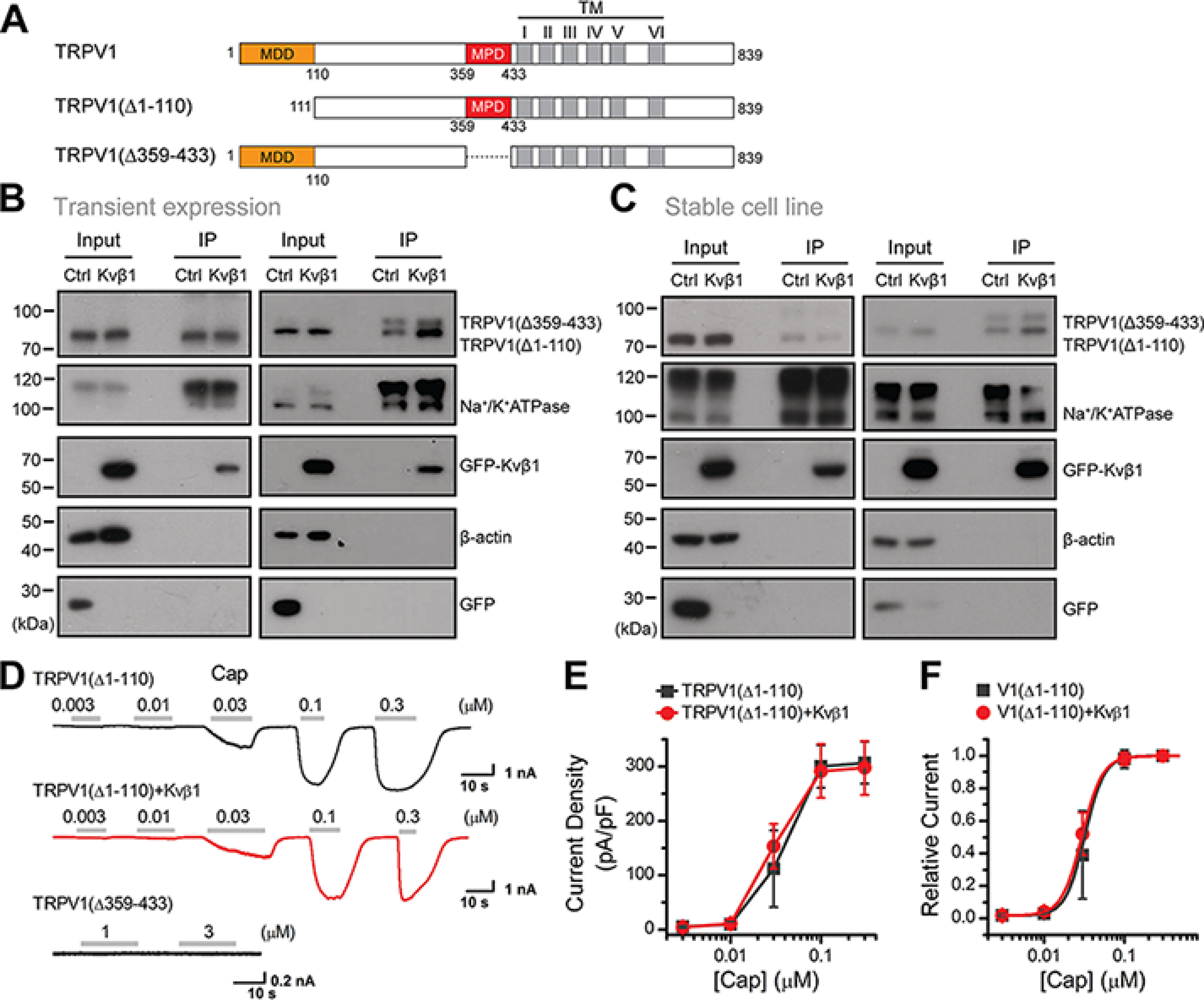
**Kvβ1 regulates the surface expression of TRPV1 by interacting with MDD of TRPV1.** *A*, schematic representation of composition of mutant channels. Residue numbers are shown. *TM*, transmembrane. *B*, HEK 293T cells were transiently transfected with the following plasmids as indicated: TRPV1(Δ1–110)-FLAG and TRPV1(Δ359–433)-FLAG together with Kvβ1-GFP or GFP. Surface level of TRPV1 were measured by IB after plasma membrane proteins were biotinylated and purified with NeutrAvidin-agarose beads. β-Actin and Na^+^/K^+^ ATPase were used as controls for cytoplasmic and membrane proteins, respectively. *C*, stable HEK 293T cell lines expressing phage-TRPV1(Δ1–110) or phage-TRPV1(Δ359–433) were transiently transfected with Kvβ1-GFP and GFP, respectively. Similar to *A*, surface levels of TRPV1 were measured by IB after plasma membrane proteins were biotinylated and purified with NeutrAvidin-agarose beads. All blot images are representative of at least three independent experiments. *D*, representative whole-cell currents evoked by increasing concentrations of capsaicin (*Cap*) for HEK 293T cells that expressed TRPV1(Δ1–110) (*top panel*), TRPV1(Δ1–110) + Kvβ1 (*middle panel*), and TRPV1(Δ359–433) (*bottom panel*). Holding potential was −60 mV. *E*, comparison of current density evoked by capsaicin between TRPV1(Δ1–110) (*n* = 6) and TRPV1(Δ1–110) + Kvβ1 (*n* = 6). Note that there were no detectable responses for TRPV1(Δ359–433) (*n* = 4). *F*, dose-response curves of capsaicin. Fitting by Hill's equation resulted in the following: EC_50_ = 0.03 ± 0.01 μm and *n*_H_ = 3.6 ± 0.2 (*n* = 6) for TRPV1(Δ1–110) and EC_50_ = 0.03 ± 0.01 μm and n_H_ = 3.4 ± 0.2 (*n* = 6) for TRPV1(Δ1–110) + Kvβ1. *Error bars* indicate S.D. Full images of the Western blots are shown in Fig. S7.

To determine the relative role of N-terminal domains in the regulation of TRPV1 surface expression level, we constructed TRPV1 truncations that lack the Kvβ1 interaction segments: TRPV1(Δ1–110) and TRPV1(Δ359–433) (Fig. 5*A*). We first transfected the FLAG-TRPV1(Δ1–110) into HEK 293T cells and 12 h later equally divided the cells into two groups that were then transfected with Kvβ1-GFP or GFP, respectively. Similar experimental procedures were performed for the FLAG-TRPV1(Δ359–433) construct. When the introduced proteins were fully expressed (*i.e.* 24 h after the last transfection), we performed surface biotinylation analysis. As shown in Fig. 5*B*, although the total amount of both truncated TRPV1 proteins was unaffected by Kvβ1, the amount of surface-expressed FLAG-TRPV1(Δ359–433) (*i.e.* comprising the MDD aa 1–110) was steadily increased. In contrast, the absence of the MDD within the FLAG-TRPV1(Δ1–110) construct rendered it irresponsive to Kvβ1 regulation.

To further explore the effect of the MDD on TRPV1 membrane targeting, we developed HEK 293T cell lines stably expressing phage-TRPV1(Δ1–110) and phage-TRPV1(Δ359–433), respectively. We then transfected Kvβ1-GFP or GFP into the stable-expression cell lines separately and conducted a surface biotinylation experiment after 24 h. As shown in Fig. 5*C*, the presence of Kvβ1 displayed no effect on both the cytoplasm and plasma membrane expression of the MDD-devoid TRPV1(Δ1–110) construct but increased the surface expression of the TRPV1(Δ359–433) construct. Thus, Kvβ1 interacts with the MDD region to regulate TRPV1 surface expression.

We also examined the effect of Kvβ1 on the electrophysiological activity of the TRPV1 mutants. Whole-cell recordings revealed that TRPV1(Δ359–433) showed no channel activity, whereas TRPV1(Δ1–110) showed current responses similar to TRPV1(Fig. 5*D*). We then explored the effect of Kvβ1 on the channel gating of TRPV1(Δ1–110). We recorded TRPV1(Δ1–110) responses to increasing concentrations of capsaicin at *V*_h_ = −60 mV. The current density showed no significant change in the presence of Kvβ1 (Fig. 5*E*), distinct from its facilitatory effect observed for WT TRPV1 (Fig. 2*G*). Fitting the normalized dose-response curves with the Hill equation yielded similar EC_50_ values and Hill coefficients (*n*_H_) for TRPV1(Δ1–110) co-expressed with Kvβ1 or not (Fig. 5*F*). This result suggests that Kvβ1 interacts with MDD to regulate TRPV1 surface expression and current response.

### Kvβ1 modulates TRPV1 temperature threshold by interacting with MPD domain

We have previously demonstrated that the N-terminal region connecting ankyrin repeats to the first transmembrane segment is critical for TRPV1 temperature sensing (20). Because the Kvβ1 analog Kvβ2 has been suggested to modulate TRPV1 activity (21), we sought to compared their effect on TRPV1 thermal activation. Kvβ2 protein sequence is 85% identical to Kvβ1 except the first 72 amino acids at the N terminus. By similar experimental procedure to explore Kvβ1-TRPV1 interactions, we observed that Kvβ2 could only be precipitated by FLAG-1–359, *i.e.* the region from the initiating N terminus of TRPV1 to the end of the ankyrin repeats, whereas the TRPV1 MPD domain (aa 359–433) domain was only precipitated by Kvβ1-FLAG (Fig. S3). Hence, distinct from Kvβ1 that interacts with MDD and MPD domains, Kvβ2 shows interaction with TRPV1 N-terminal 1–359 segment.

**Figure 6.**
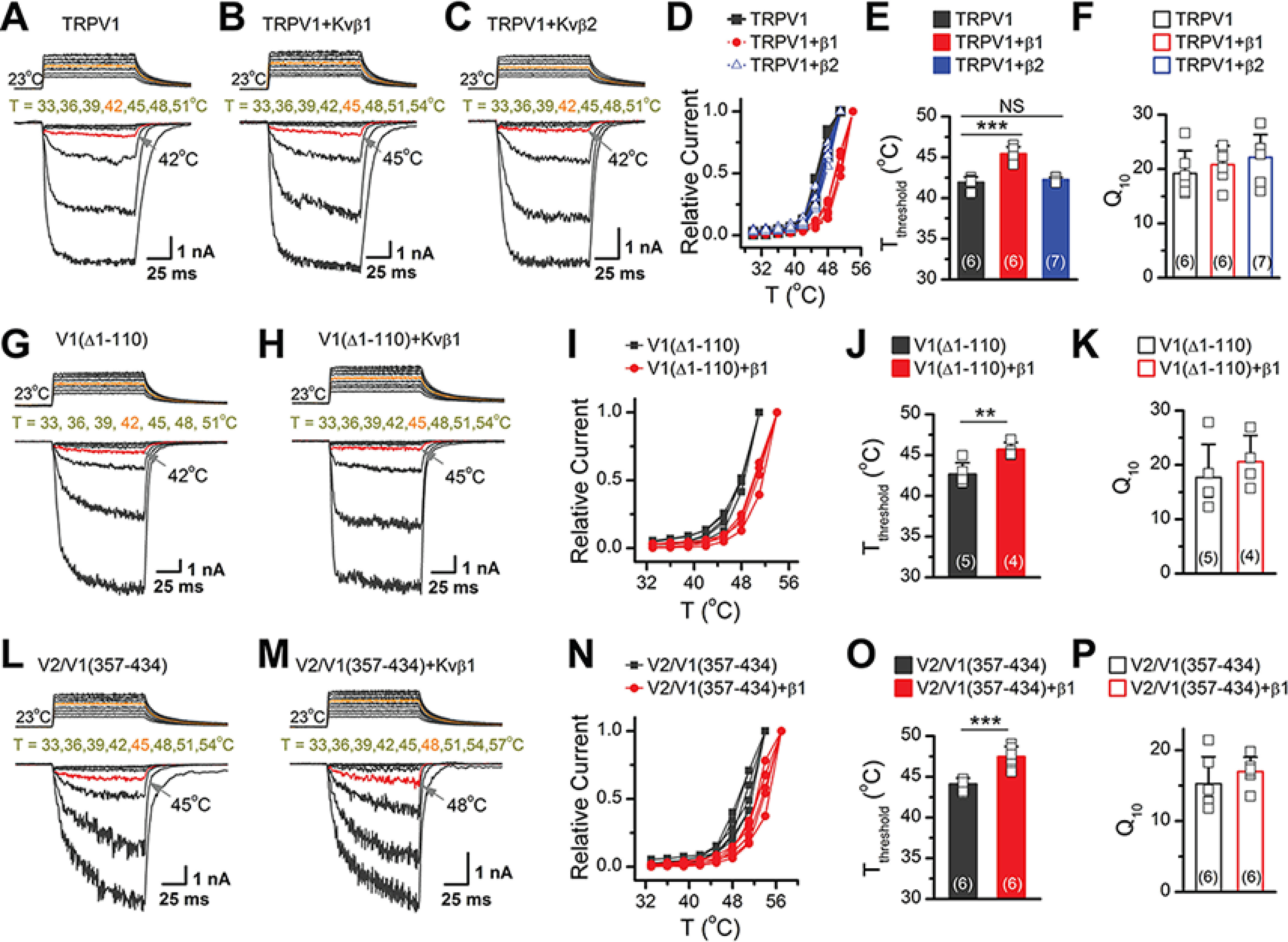
**Kvβ1 modulates temperature threshold for TRPV1 activation by interacting with MPD of TRPV1.** *A–C*, representative responses to a family of temperature jumps for HEK 293T cells that expressed TRPV1 (*A*), TRPV1 + Kvβ1 (*B*), and TRPV1+ Kvβ2 (*C*). Temperature pulses stepped from room temperature were generated by local laser irradiation were 100 ms long and had a rise time of 1.5 ms. Temperatures were calibrated offline from the pipette current using the temperature dependence of electrolyte conductivity (see “Experimental Procedures”). The *red traces* indicate the temperature threshold for heat activation of TRPV1. *D*, temperature dependence of steady-state response. Each curve represents measurements from an individual cell, and the responses were normalized to its maximum responses. *E*, comparison of temperature threshold for activation of TRPV1. Different *symbols* represent individual data points. The mean temperature thresholds (*T*_threshold_) of activation were 41.9 ± 0.7 °C for TRPV1 (*n* = 6), 45.4 ± 0.8 °C for TRPV1 + Kvβ1 (*n* = 6), and 42.3 ± 0.3 °C for TRPV1 + Kvβ2 (*n* = 7), respectively. *P* < 0.001 for *T*_threshold_ of TRPV1 *versus* TRPV1 + Kvβ1 and *P* = 0.37 for *T*_threshold_ of TRPV1 *versus* TRPV1 + Kvβ2 by one-way ANOVA test. *F*, comparison of temperature coefficient. The values of *Q*_10_ derived from the linear fits in Arrhenius plot of steady-state currents were as following, *Q*_10_ = 19.2 ± 4.2 for TRPV1 (*n* = 6), *Q*_10_ = 20.8 ± 3.5 for TRPV1 + Kvβ1 (*n* = 6), and *Q*_10_ = 22.2 ± 4.2 for TRPV1 + Kvβ2 (*n* = 7). *Colored symbols* indicate individual data points. *P* = 0.49 for *Q*_10_ of TRPV1 *versus* TRPV1 + Kvβ1 and *P* = 0.20 for *Q*_10_ of TRPV1 *versus* TRPV1 + Kvβ2 by one-way ANOVA test. *G* and *H*, similar recordings in HEK 293T cells that expressed TRPV1(Δ1–110) (*G*) and TRPV1(Δ1–110) + Kvβ1 (*H*). *I*, temperature-response curves for TRPV1(Δ1–110) and TRPV1(Δ1–110) + Kvβ1. *J*, summary plot of threshold changes. *T*_threshold_ = 42.6 ± 1.4 °C for TRPV1(Δ1–110) alone (*n* = 5) and *T*_threshold_ = 45.7 ± 0.9 °C for TRPV1(Δ1–110) + Kvβ1 (*n* = 4). *P* = 0.008 by Student's *t* test. *K*, comparison of *Q*_10_. *Q*_10_ = 17.7 ± 6.1 for TRPV1(Δ1–110) alone (*n* = 5), *Q*_10_ = 20.6 ± 4.8 for TRPV1(Δ1–110) + Kvβ1 (*n* = 4). *P* = 0.47. *L* and *M*, parallel recordings of heat responses in HEK 293T cells transfected with TRPV2/TRPV1(357–434) and TRPV2/TRPV1(357–434) + Kvβ1. *N*, temperature-response curves for TRPV2/TRPV1(357–434) and TRPV2/TRPV1(357–434) + Kvβ1. *O* and *P*, comparison of temperature threshold and Q10, respectively. *T*_threshold_ = 44.1 ± 0.8 °C for TRPV2/TRPV1(357–434) alone (*n* = 6), *T*_threshold_ = 47.4 ± 1.3 °C for TRPV2/TRPV1(357–434) + Kvβ1 (*n* = 6). *P* = 0.0002. *Q*_10_ = 15.3 ± 3.8 for TRPV2/TRPV1(357–434) alone (*n* = 6), and *Q*_10_ = 17.0 ± 2.0 for TRPV2/TRPV1(357–434) + Kvβ1 (*n* = 6). *P* = 0.35. Different *symbols* represent individual recordings. *Error bars* indicate S.D.

We then comparatively studied the effect of Kvβ1 and Kvβ2 binding on TRPV1 temperature sensing. Using laser irradiation-based temperature controlling and whole-cell patch-clamp recording, we recorded current responses in three groups of HEK 293T cells expressing TRPV1 alone, TRPV1 with Kvβ1 or Kvβ2, respectively. The presence of Kvβ2 showed no effect on the temperature threshold (*T*_threshold_); currents started to be induced at 42 °C as observed for TRPV1 alone. In contrast, cells expressing TRPV1 and Kvβ1 did not exhibit significant responses until 45 °C (Fig. 6, *A–C*). An appreciable right shift of the temperature dependence curve of TRPV1 was observed with Kvβ1, but not Kvβ2 (Fig. 6*D*). The expression of Kvβ1 resulted in a rise of ∼3°C in TRPV1 *T*_threshold_, without affecting the temperature coefficient (*Q*_10_), whereas Kvβ2 showed no effect (Fig. 6, *E* and *F*).

Our domain mapping analysis showed that Kvβ1 interacted with both MDD and MPD of TRPV1 channel (Fig. 4). To examine their involvement in Kvβ1-induced *T*_threshold_ change, we used the truncated TRPV1(Δ1–110) construct that is specifically devoid of the MDD. Co-expression of Kvβ1 still elevated the *T*_threshold_ by ∼3°C (Fig. 6, *G–K*), indicating the dispensable role of MDD. Further we generated the chimera construct TRPV2/TRPV1(357–434) that introduced specifically the TRPV1 MPD domain into the TRPV2 channel. This engineering rendered the chimera channel with an activation *T*_threshold_ of ∼45 °C, which is consistent with the previous report (20). With the insertion of the TRPV1 MPD, we also observed that the *T*_threshold_ of TRPV2/TRPV1(357–434) was raised by ∼3°C when co-expressed with Kvβ1 (Fig. 6, *L–P*). Taken together, these results suggest that Kvβ1 interacts with MPD domain to regulate TRPV1 temperature sensitivity.

## Discussion

As a polymodal sensor, TRPV1 ion channel responds to a variety of exogenous stimuli and is implicated in pain sensing and transduction (2, 22–24). In addition to being regulated at the channel level, TRPV1 function is also controlled by multiprotein signaling assemblies (25, 26). Illustrating the molecular components of the TRPV1 signal complexes helps to understand its function in physiopathological conditions.

**Figure 7.**
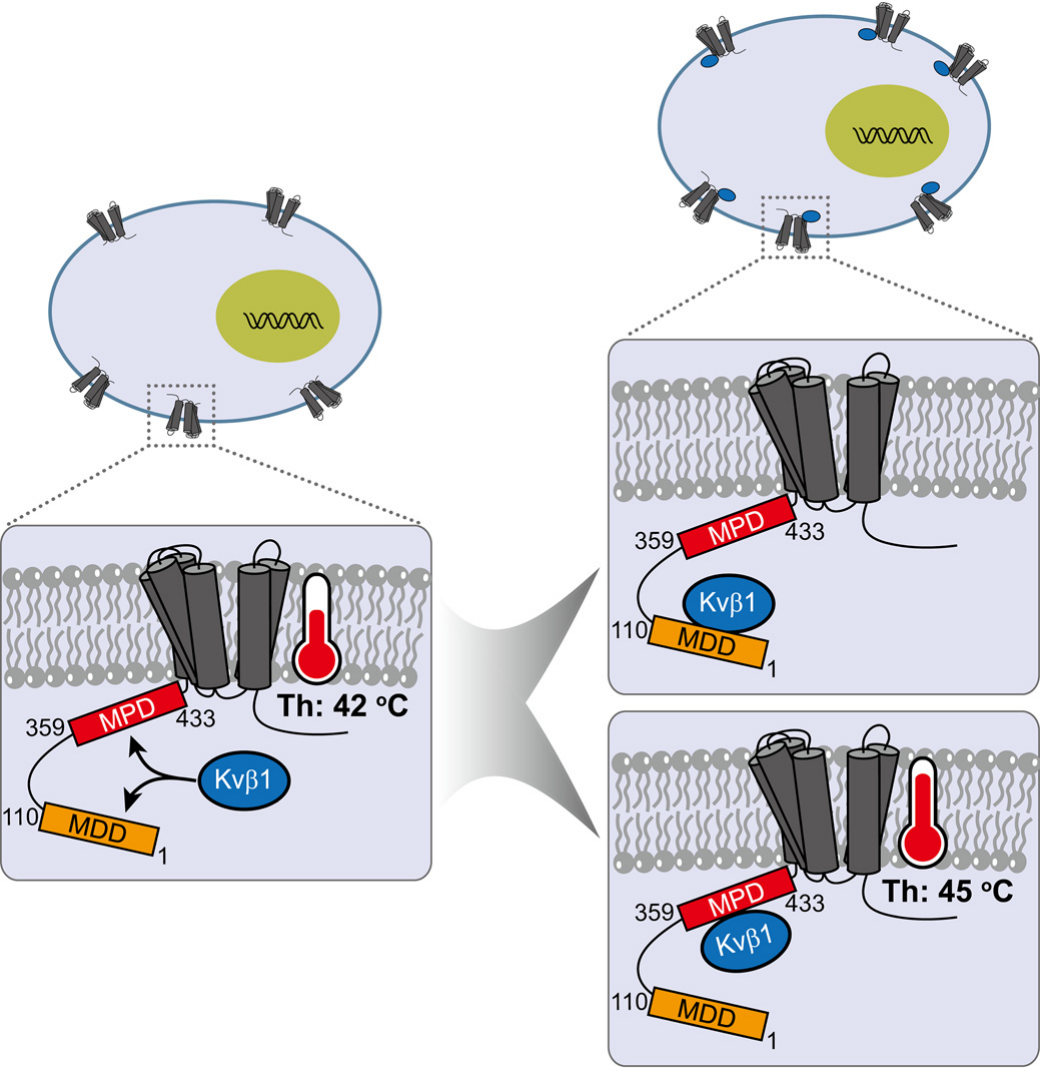
**Schematic model of the dual regulatory mechanism of Kvβ1.** Two discrete domains at N terminus of TRPV1 are responsible for the interaction between TRPV1 and Kvβ1. Mechanistically, Kvβ1 interacts with the MDD (aa 1–110) and MPD (aa 359–433) of TRPV1 to respectively regulate its surface expression and temperature-activation threshold, respectively.

We here demonstrate that two discrete domains at N terminus of TRPV1 are responsible for the interaction between TRPV1 and Kvβ1. By site-specific mapping, we revealed that Kvβ1 interacted with the membrane-distal domain (MDD, aa 1–110) and membrane-proximal domain (MPD, aa 359–433) of TRPV1 to, respectively, regulate its surface expression levels and temperature-activation threshold by 3 °C. These data suggest that Kvβ1 is a molecular element in the TRPV1 signaling complex and exerts site-specific dual regulatory effects (Fig. 7).

Previous studies have shown that Kvβ1 regulates Shaker-like potassium channels (*e.g.* Kv1) by the interaction with the N-terminal A and B box domain of the Shaker family channels (27). Kvβ subunits had also been found to increase the expression of Kv4.3 channels by interacting with the C termini (28). Here we show that the surface expression of TRPV1 is enhanced by the interaction of Kvβ1 with the N-terminal MDD. We, however, found no homologous sequences among the N terminus of TRPV1, Kv1 channel, and C terminus of Kv4.3 channels, suggesting a specific regulatory effect of Kvβ1 on TRPV1. Here we observed that Kvβ1 facilitates the surface expression of TRPV1, thereby up-regulating whole-cell current density. Kvβ1 may have a similar regulatory mechanism for potassium channels (28–30), an issue requiring additional investigations.

As a Kvβ1 analog, Kvβ2 has been suggested to influence TRPV1 expression (21). Different regulatory effects have been noted for these two subunits. For instance, Kvβ1, but not Kvβ2, confers rapid A-type inactivation to noninactivating Kv1 channels (18). Here, we observed that Kvβ1 regulates TRPV1 functions in a manner distinct from Kvβ2. Kvβ1 interacts with both MDD and MPD regions of TRPV1, whereas Kvβ2 interacts with the aa 1–359 segment. Notably, the thermal activation threshold of TRPV1 was up-regulated by ∼3°C by the presence of Kvβ1, an effect attributed to the interaction with TRPV1 MPD domain and not seen with Kvβ2. This result confirms the importance of the MPD domain in setting the thermal activation property of TRPV1 (20). It should be noted that under the condition of exogenous expression, Kvβ1 elevates the TRPV1 *T*_threshold_ to ∼45°C, whereas it remains at 42 °C in endogenous DRG neurons. It seems that a relatively high amount of Kvβ1 is required to change TRPV1 temperature threshold, which might not be the case for DRG neurons. Another possibility is that although Kvβ1 tends to increase TRPV1 thermal activation threshold, there are other mechanisms to counterbalance the effect of Kvβ1 in sensory neurons. Notwithstanding, our results unveil an important role for Kvβ1 in sculpturing TRPV1 thermal responses.

In 13-lined ground squirrels and Bactrian camels, changing a single amino acid raised the TRPV1 activation threshold to ∼46°C or even higher (31). The increased heat tolerance may prove beneficial by conferring animals the ability to inhabit otherwise prohibitive ecological niches. Thus, modulation of the TRPV1 temperature-activation threshold by Kvβ1 might have physiological implications. Targeting the Kvβ1-TRPV1 interaction would help to tune the thermal reactions along the body sensory input pathways.

## Experimental procedures

### Constructs, cell culture, and transfection

The WT rat TRPV1 cDNA was a gift from Dr. Feng Qin (State University of New York at Buffalo, Buffalo, NY, USA). The full lengths of mouse Kvβ1 and Kvβ2 were obtained and subcloned into pEGFP-N1 vector, respectively. To express in mammalian cells, the cDNAs of TRPV1, Kvβ1, and Kvβ2 were introduced into p3×FLAG-cmv-7.1, pEGFP-N1, or pcDNA5-HA, as indicated. To express GST-tagged protein of TRPV1 N terminus in prokaryotic cells, the open-reading frame of the TRPV1 N-terminal cDNA was placed into the pGEX-6P-1 vector. All recombinant constructs and mutations were carried out using the overlap-extension PCR method. The resulting constructs and mutations were then verified by DNA sequencing. Oligonucleotide DNAs targeting Kvβ1 were synthesized, annealed, and inserted into pLenti-GFP vector. The sequences of Kvβ1 shRNA are as follows: #1, 5´-GCTTGGTCATCACAACCAA-3´; #2, 5´-GATGTGGTCTTTGCAAATC-3´; and #3, 5´-GGAGTTGGTGCAATGACAT-3´. HEK 293T and ND7/23 cells were grown in Dulbecco's modified Eagle's medium (Thermo Fisher Scientific) containing 4.5 mg/ml glucose, 10% heat-inactivated fetal bovine serum (Gibco, Thermo Fisher Scientific), 50 units/ml penicillin, and 50 μg/ml streptomycin and were incubated at 37 °C in a humidified incubator gassed with 5% CO_2_. HEK 293T cells stably expressing phage-TRPV1, phage-TRPV1(Δ1–110), and phage-TRPV1(Δ359–433) were used in this study. To generate 293T/phage-TRPV1, 293T/phage-TRPV1(Δ1–110), and 293T/phage-TRPV1(Δ359–433) cells, HEK 293T cells were transiently transfected with the indicated construct using Lipofectamine 2000 (Invitrogen). Transfected cells were grown for 2 weeks in puromycin to select for stable expressing cells. Nontransfected cells were killed by the addition of puromycin (2 μg/ml) 2 days after treatment. The medium was replaced every 2–3 days until single colonies were formed, and then the stable cell lines were placed in a complete medium containing 1 μg/ml puromycin. In another set of experiments, cells grown into ∼80% confluence were transiently transfected with the desired DNA constructs using either the standard calcium phosphate precipitation method or Lipofectamine 2000 (Invitrogen) following the protocol provided by the manufacturer. In the membrane separation experiment, we transfected HEK 293T cells with the same amount (3 μg) of TRPV1 cDNA in all four groups, whereas the amount of Kvβ1 cDNA was adjusted to achieve a molar ratio of TRPV1 and Kvβ1 at 1:1 and 1:2, respectively. For the membrane separation experiment in ND7/23 cells, we transfected Kvβ1-GFP in amounts of 0, 1, 2, and 4 μg, respectively. Transfected cells were reseeded on 12-mm round glass coverslips coated by poly-l-lysine. Experiments took place usually 12–24 h after transfection.

### Isolation of DRG neurons

All mice were housed in the specific pathogen-free animal facility at Wuhan University, and all animal experiments were in accordance with protocols approved by the Institutional Animal Care and Use Committee of Wuhan University. DRG neurons were prepared as previously described with minor modifications (13). Briefly, 6–8-week-old adult C57BL/6 male mice were deeply anesthetized and decapitated. L3–L4 DRGs were isolated from the spinal column. After removing the attached nerves and surrounding connective tissues, DRG neurons were rinsed with ice-cold PBS. Then total RNA was extracted from the intact DRG for yeast cDNA library construction using TRIzol (Life Technologies) per the manufacturer's instructions or directly homogenized DRG neurons for immunoprecipitation experiments.

### Yeast two-hybrid assay

Yeast two-hybrid screen was conducted using the Matchmaker GAL4-based two-hybrid system (Clontech). The mouse DRG cDNA library was fused to the GAL4 activation domain of plasmid pGADT7 (Clontech). The titer of the primary cDNA library was calculated using the number of clones on plates. Colony PCR was used to confirm the size of the inserted fragments in the library. Bait plasmids were constructed by introducing the N terminus (aa 1–433) or C terminus (aa 688–839) of TRPV1 into the GAL4 DNA-binding domain of the pGBKT7 vector (Clontech). The pGBK bait was transformed into yeast strain Y187, and pGAD prey was expressed in AH109. Cytotoxicity and self-activation activity were determined via observing yeast clone growth and size. Diploid yeast cells from yeast mating were selected on the triple dropout medium (SD/−His/−Leu/−Trp). Then replica plate colonies were transferred onto quadruple dropout medium (SD/−Ade/−His/−Leu/−Trp) containing X-α-gal (4 mg/ml). X-α-gal was used as substrate for colorimetric detection of α-Galactosidase activity. The plates were incubated at 30 °C for 7 days. Plasmids extracted from the positive clones were transformed into *Escherichia coli* cloning host DH5α to be amplified, and samples were then sequenced individually. BLAST comparisons and other bioinformatics methods were applied for sequence analysis. Thereafter, the bait and prey plasmids in different combinations were sequentially co-transformed into yeast stain AH109 and selected on double dropout medium (SD/−Leu/−Trp) and incubated at 30 °C for 3–4 days. Then the positive clones were diluted and equally coated onto SD/−Leu/−Trp/−His/−Ade medium with X-α-gal and SD/−Leu/−Trp medium and cultured at 30 °C for 3–4 days. In parallel, the combination of pGBKT7–53/pGADT7–T and pGBKT7–Lam/pGADT7–T were used as positive and negative controls, respectively.

### Immunoprecipitation and immunoblotting assays

Denaturing immunoprecipitation was performed as previously described (9). Briefly, HEK 293T or ND7/23 cells were harvested 24 h post-transfection and solubilized in Nonidet P-40 lysis buffer (20 mm Tris-HCl, pH 7.4, 150 mm NaCl, 1 mm EDTA, 1% Nonidet P-40) containing 1% protease inhibitor mixture. Cell lysates were subjected to SDS-PAGE, and immunoblot analysis was performed with the appropriate antibodies. For immunoprecipitation assays, the solubilized supernatants were incubated with the control IgG or specific antibodies and protein G–agarose beads for 2 h. Immunocomplexes were washed three times with prelysis buffer (20 mm Tris-HCl, pH 7.4, 150 mm NaCl, 1 mm EDTA, 1% Triton X-100) containing 0.5 m NaCl, followed by immunoblot analysis.

### GST pulldown assay

GST-fusion protein GST-Nt-TRPV1 or GST alone were expressed in BL21 competent cells that were induced with isopropyl β-d-thiogalactopyranoside (1 mm) at 16 °C for 18 h, and the proteins were purified using GSH-Sepharose 4B (Transgen Biotech, Beijing, China). HEK 293T cells were transiently transfected with FLAG-tagged Kvβ1 (Kvβ1-FLAG) cDNA and cultured for 24 h. GST-Nt-TRPV1 or GST were incubated with HEK 293T extracts that expressing Kvβ1-FLAG at 4 °C overnight in Nonidet P-40 lysis buffer containing protease inhibitors. The precipitates were washed three times with the prelysis buffer containing 0.5 m NaCl, followed by immunoblot analysis.

### Surface biotinylation assay

Surface biotinylation was performed following the established protocols (9). Cells that expressed TRPV1-FLAG and Kvβ1-GFP (or GFP as control) were washed three times with ice-cold PBS (pH 8.0) supplemented with 1 mm MgCl_2_ and 2.5 mm CaCl_2_. Then the sulfo-NHS-LC-Biotin (Thermo Fisher Scientific) was added to the same solution at 0.25 mg/ml and incubated with cells for 25 min at 4 °C with gentle rocking. The cells were rinsed with PBS containing 0.1 m glycine for 20 min at 4 °C to quench unbounded biotin. Biotin-labeled proteins were isolated by incubating whole-cell lysates with NeutrAvidin-agarose beads (Thermo Fisher Scientific) overnight at 4 °C with rocking. The beads were washed three times with PBS (pH 8.0), and bound proteins were eluted with the boiling SDS sample buffer and used for immunoblotting.

### Antibodies and chemicals

Rabbit anti-TRPV1 (Alomone laboratory, catalog no. ACC-030), mouse anti-Kvβ1 (Santa Cruz, catalog no. sc-373986), rabbit anti-Na^+^/K^+^ ATPase (Abcam, catalog no. ab76020), mouse anti-GST (Abclonal, catalog no. AE001), mouse anti-β-actin (Transgen Biotech, catalog no. 6609-1), mouse anti-FLAG (Sigma, catalog no. F3165), mouse anti-FLAG (MBL, catalog no. M185-3L), rabbit anti-FLAG (Proteintech, catalog no. 20543-1-AP), mouse anti-HA (MBL, catalog no. M180-3), rabbit anti-HA (Earthox, catalog no. E02218002), mouse anti-GFP (Tianjin Sungene Biotech, catalog no. KM8009), rabbit anti-GFP (WB: Solarbio, catalog no. B1025F), anti-FLAG affinity gel (Bimake, catalog no. B23100), anti-HA affinity gel (Bimake, catalog no. B23301), ProteinIso GST resin (Transgen Biotech, catalog no. DP201-01), goat anti-mouse IgG (H + L) (Jackson Immunoresearch, catalog no. 115-035-003), and goat anti-rabbit IgG (H + L) (Jackson Immunoresearch, catalog no. 111-005-003) were purchased from the indicated manufactures. The EZ-Link Sulfo-NHS-LC-Biotin (catalog no. 21335) and high-capacity NeutrAvidin-agarose (catalog no. 29202) were ordered from Thermo Fisher Scientific.

### Electrophysiology

Conventional whole-cell patch-clamp recording methods were used. For the recombinant expressing system, green fluorescence from GFP was used as a marker for gene expression. Patch-clamp recordings were voltage clamped using an EPC10 amplifier (HEKA, Lambrecht, Germany). Voltage commands were made from the Patchmaster program. For a subset of recordings, currents were amplified using an Axopatch 200B amplifier (Molecular Devices, Sunnyvale, CA, USA) and recorded through a BNC-2090/MIO acquisition system (National Instruments, Austin, TX, USA) using QStudio developed by Dr. Feng Qin at State University of New York at Buffalo. Recording pipettes were pulled from borosilicate glass capillaries (World Precision Instruments) and fire-polished to a resistance of 2–4 MΩ when filled with electrode saline containing 140 mm CsCl, 5 mm EGTA, and 10 mm HEPES, pH 7.4, adjusted with CsOH, and the bath solution for whole-cell recording consisted of 140 mm NaCl, 5 mm KCl, 3 mm EGTA, and 10 mm HEPES, pH 7.4, adjusted with NaOH. HEPES was used for pH 7.0–7.4, and instead of HEPES, MES was used as the pH buffer when pH < 6.5. For recordings under low pH conditions, 50 μm amiloride was also included to inhibit native acid–sensing ion channels. Exchange of external solutions was performed using a gravity-driven local perfusion system. As determined by the conductance tests, the solution around the patched cell was fully controlled by the application of a solution with a flow rate of 100 μl/min or greater. For voltage-dependence experiments, a voltage step protocol consisting of 200-ms depolarizing pulses ranging from −100 to 200 mV with 20-mV increments was triggered from the holding potential of −60 mV. The compensation of pipette series resistance and capacitance were taken by using the circuitry of the amplifier (> 80%) to reduce voltage errors. Capsaicin was dissolved in pure ethanol to make a stock solution and diluted in the bath solution to the desired concentrations right before the experiments. The final concentrations of ethanol did not exceed 0.3%, which had no effect on the currents. Whole-cell recordings were sampled at 5 kHz and filtered at 1 kHz. Unless otherwise noted, all chemicals were purchased from Sigma (Millipore–Sigma). All patch-clamp recordings except heat activation were made at room temperature (22–24°C).

### Ultrafast temperature jump achievement

A rapid temperature jump system is produced by using a single emitter IR laser diode (1470 nm) as previously described (32). Briefly, a multimode fiber with a core diameter of 100 μm was used to transmit the launched laser beam. The other end of the exposed fiber core was placed close to the cell of interest where the perfusion pipette is typically positioned. The laser diode was driven by a pulsed quasi-CW current power supply (Stone Laser, Beijing, China), and the pulsing of the controller was controlled from a computer through the data acquisition card (National Instruments), which was also responsible for patch-clamp recordings. A blue laser line (460 nm) was coupled into the same fiber to aid alignment. Constant temperature steps were generated by irradiating the tip of an open pipette filled with the pipette solution and using the current through the electrode as a readout for feedback control. The laser diode was first powered on for a brief duration to get the target temperature and was then modulated to achieve a constant pipette current. The profile of the modulation pulses was stored and subsequently played back to apply the temperature jump to the cell of interest. Temperature was calibrated offline from the electrode current based on the temperature dependence of electrolyte conductivity. The threshold temperature for heat activation of TRPV1 was determined as the temperature at which the slow inward current (∼10% of its maximum response) was evoked.

### Statistical analysis

The data were analyzed offline with Clampfit (Molecular Devices), IGOR (Wavemetrics, Lake Oswego, OR, USA), SigmaPlot (SPSS Science, Chicago, IL, USA), OriginPro (OriginLab Corporation, MA, USA), and ImageJ (National Institutes of Health). Conductance–voltage (*G*–*V*) curves for activation were derived from steady-state currents, converted to conductance, and then fitted by Boltzmann function: *G*/*G*_max_ =1/(1 + exp((*V* − *V*_1/2_)/κ)), in which *G*_max_ is the maximal conductance, *V*_1/2_ is the voltage at which the conductance (*G*) is half the maximum conductance (*G*_max_), and κ is the slope factor. For dose-response analysis, the modified Hill equation was used: Y = A1 + (A2−A1)/(1 + (EC_50_/[toxin])^nH^), in which *n*_H_ is the Hill coefficient. All data are expressed as means ± S.D., with statistical significance assessed using unpaired Student's *t* test for two-group comparison. Fig. 6 (*E* and *F*) were analyzed with one-way analysis of variance (ANOVA) test for multiple group comparison. Significant difference is indicated by *p* values less than 0.05: *, *p* < 0.05; **, *p* < 0.01; and ***, *p* < 0.001.

## Data availability

The data that support the findings of this study are available from the corresponding author upon reasonable request.

## Supplementary Material

Supporting Information
