# Supplementary material for: Site-specific contacts enable distinct modes of TRPV1 regulation by the potassium channel Kvβ1 subunit
Source: J Biol Chem. 2020 Oct 15;295(50):17337–48. doi: 10.1074/jbc.RA120.015605 (PMC7863878; doi:10.1074/jbc.RA120.015605)
Supplement: Supporting Information [file supp_295_50_17337__index.html]

Site-specific contacts enable distinct modes of TRPV1 regulation by the potassium channel Kvβ1 subunit — Dual regulation of TRPV1 by Kvβ1 — Site-specific contacts enable distinct modes of TRPV1 regulation by the potassium channel Kvβ1 subunit — Dual regulation of TRPV1 by Kvβ1 — Supporting Information 

# Site-specific contacts enable distinct modes of TRPV1 regulation by the potassium channel Kvβ1 subunit

## Supporting Information

- Supporting Information (to be published online) - Supporting Information-clean version
