# Supplementary material for: Site-specific contacts enable distinct modes of TRPV1 regulation by the potassium channel Kvβ1 subunit
Source: J Biol Chem. 2020 Oct 15;295(50):17337–48. doi: 10.1074/jbc.RA120.015605 (PMC7863878; doi:10.1074/jbc.RA120.015605)
Supplement: Supporting Information [file supp_RA120.015605_162867_2_supp_611481_qn53w4.docx]

**Supporting Informations**

**Site-specific contacts enable distinct modes of TRPV1 regulation by the potassium channel Kvβ1 subunit**

Yuanyuan Wang^1,3^, Xiaoyi Mo^1,3^, Conghui Ping^1^, Qian Huang^1^, Hao Zhang^1^, Chang Xie^1^, Bo Zhong^1^, Dongdong Li^2^, Jing Yao^1🖂^

^1^State Key Laboratory of Virology, Hubei Key Laboratory of Cell Homeostasis, College of Life Sciences, Frontier Science Center for Immunology and Metabolism, Wuhan University, Wuhan, Hubei 430072, China

^2^Sorbonne Université, Institute of Biology Paris Seine, Neuroscience Paris Seine, CNRS UMR8246, INSERM U1130, Paris 75005, France

^3^These authors contributed equally to this work.

🖂 Corresponding author: Jing Yao

Email: [jyao@whu.edu.cn](mailto:jyao@whu.edu.cn)

**Running title:** Dual regulation of TRPV1 by Kvβ1

**Keywords**: Kvβ1 subunit, Nociception, Protein-protein interaction, TRPV1, Pain, Thermal sensation


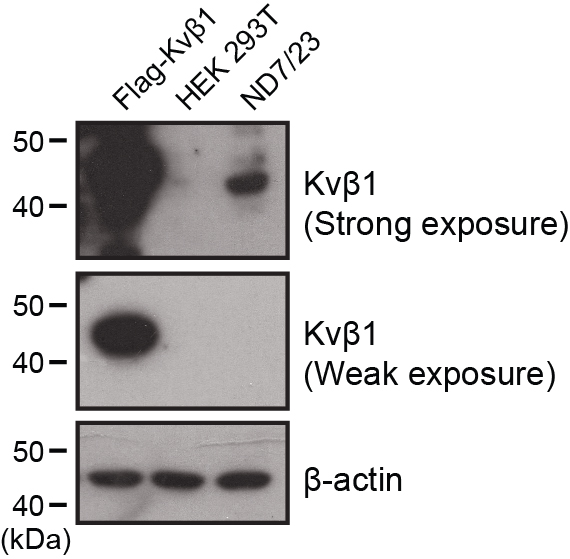


**Fig. S1. Expression levels of endogenous Kvβ1 in HEK 293T and ND7/23 cells.** Immunoblot analysis of Kvβ1 expression levels in HEK 293T cells that were transfected with Flag-Kvβ1, blank HEK 293T cells and ND7/23 cells. Films were taken under strong or weak film-exposure times. Protein molecular weight standards (in kDa) are indicated on the left side.


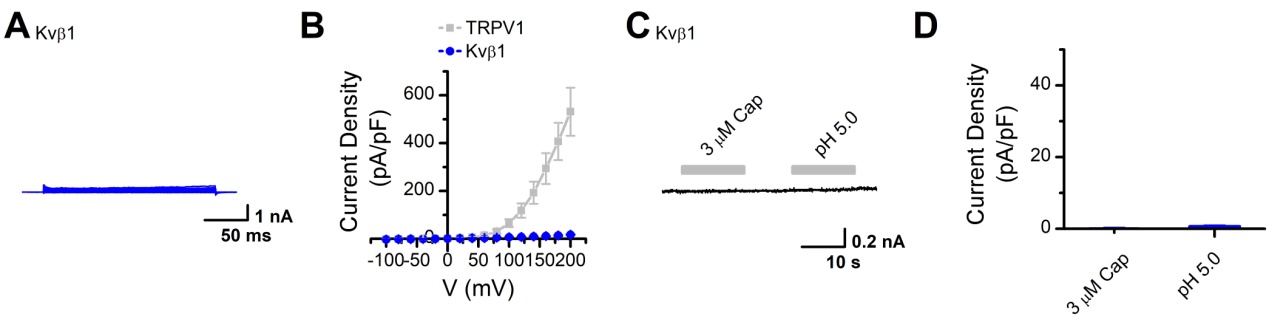


**Fig. S2. No detectable responses to voltage, capsaicin or low pH stimulation in Kvβ1-expressing HEK 293T cells.** (**A**) Whole-cell recordings were used to record currents in HEK 293T cells that expressed Kvβ1 alone. Currents were elicited with 200-ms test pulses ranging from -100 mV to +200 mV in a 20-mV increment. Initial holding potential was -60 mV. (**B**) Comparison of current density between HEK 293T cells expressing only TRPV1 or Kvβ1. (**C**) No currents evoked by 3 μM capsaicin or pH 5.0 in HEK 293T cells that expressed Kvβ1 alone. (**D**) Summary of current responses of HEK 293T cells that expressed only Kvβ1.

**
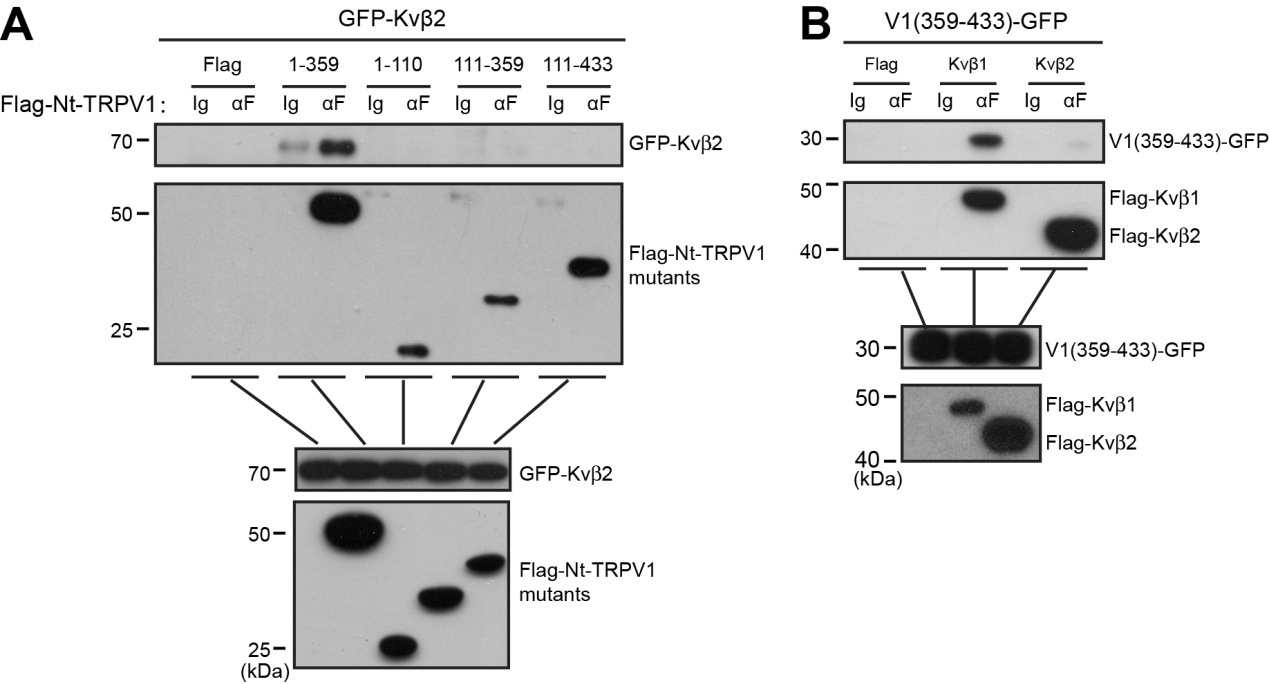
**

**Fig. S3. Mapping the interaction domains between Kvβ2 and the N-terminus of TRPV1.** (**A**) The truncation mutants of N-terminus of TRPV1 that were studied included 1-110, 1-359, 111-359 and 111-433 segments, respectively, were tagged with 3xFlag and co-transfected with Kvβ2-GFP in HEK 293T cells. Cell lysates were immunoprecipitated with anti-Flag agarose beads and analyzed by immunoblotting (IB) using anti-GFP and anti-Flag, respectively. Whole-cell lysates were also used for IB with anti-GFP and anti-Flag as input. (**B**) V1(359-433)-GFP was expressed in HEK 293T cells, together with Kvβ1-Flag, Kvβ2-Flag or Flag vector. Cell lysates were subjected to IP by anti-Flag, followed by IB using anti-GFP and anti-Flag antibodies, respectively. Whole-cell lysates were also used for IB with anti-GFP and anti-Flag as input. Expression levels of TRPV1(359-433)-GFP were similar in all cases.


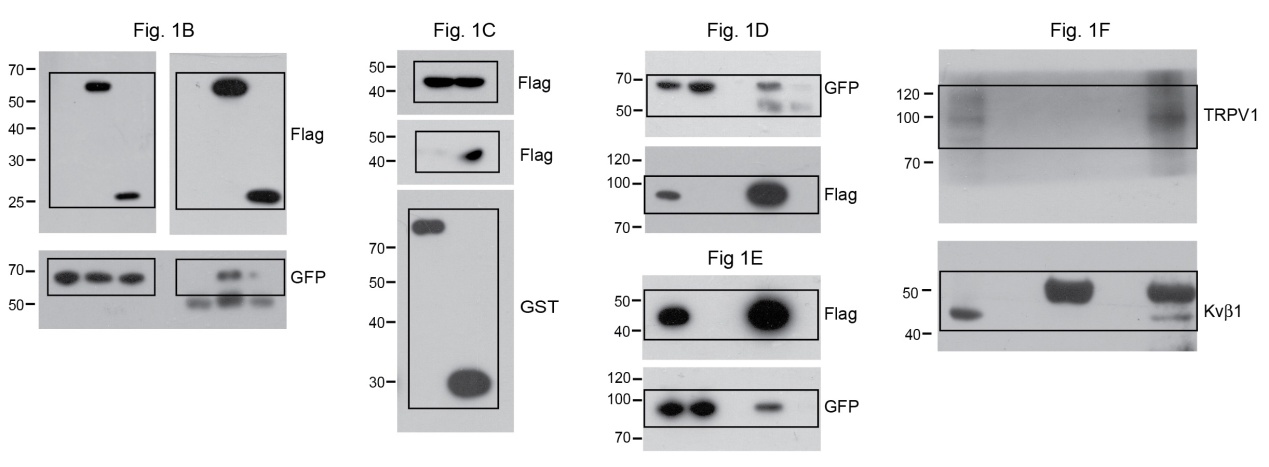
**Fig. S4. Full images of western blots of Figure 1.**


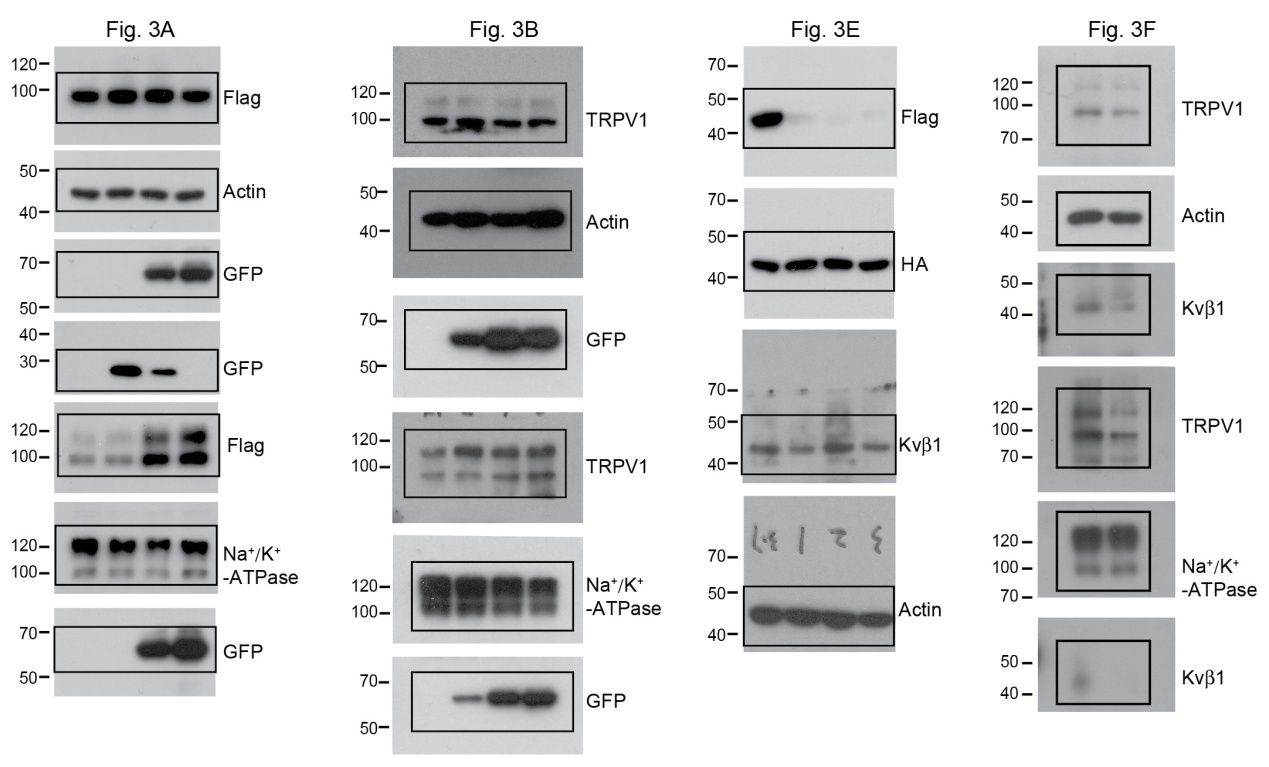


**Fig. S5. Full images of western blots of Figure 3.**


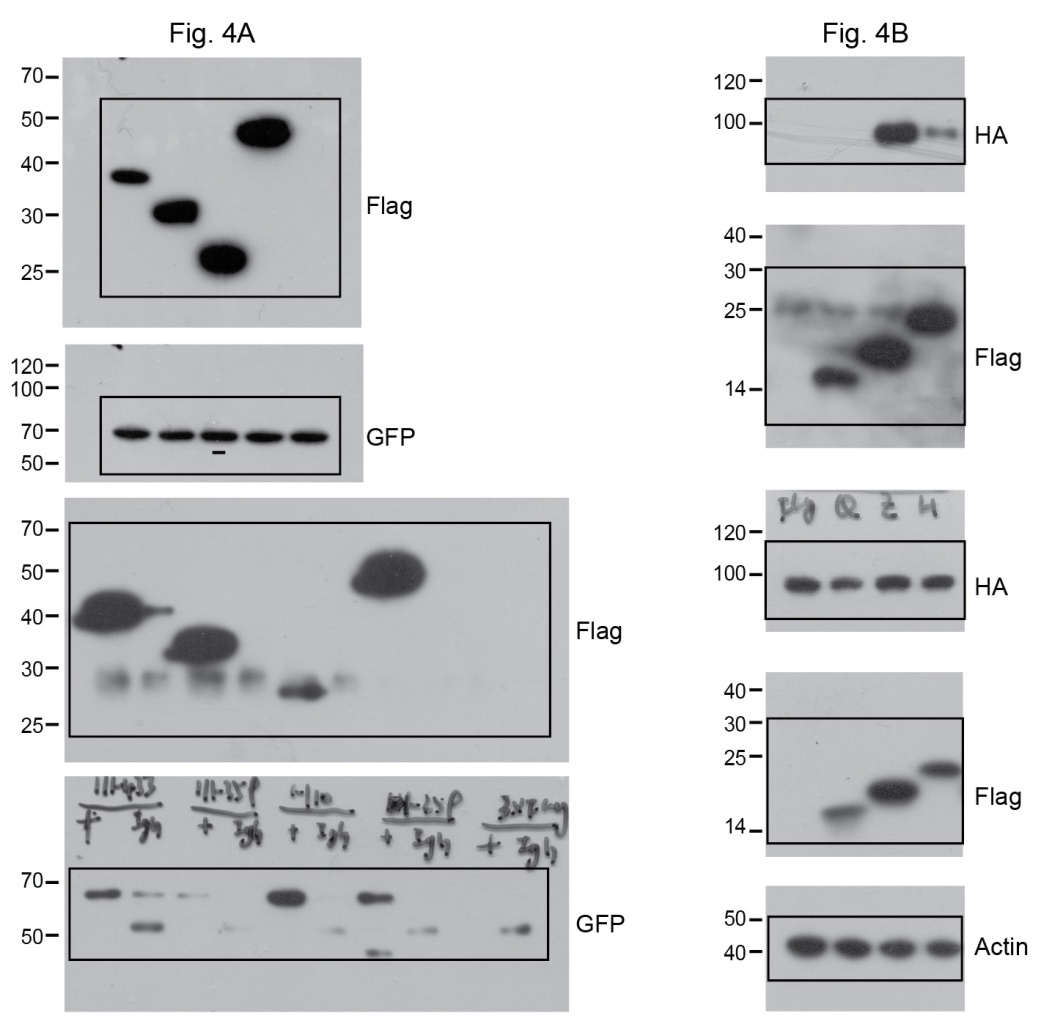


**Fig. S6. Full images of western blots of Figure 4.**


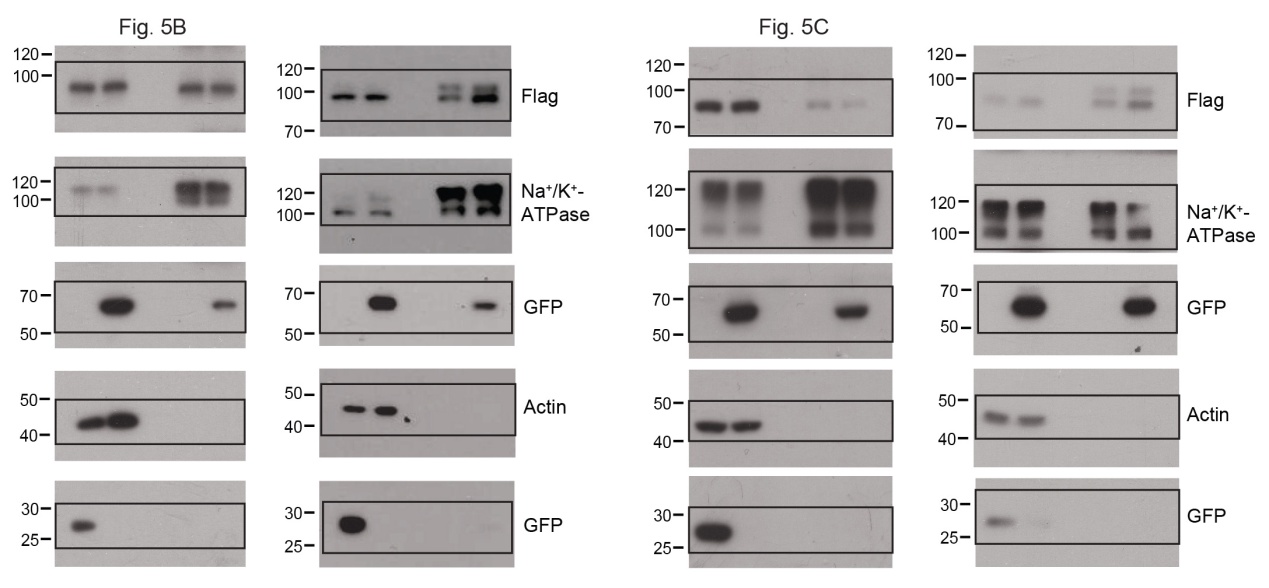


**Fig. S7. Full images of western blots of Figure 5.**
